# Supplementary material for: Application of the CPER reverse genetics system for genetic engineering of rabies virus
Source: J Virol. 2026 Mar 13;100(4):e01872-25. doi: 10.1128/jvi.01872-25 (PMC13098199; doi:10.1128/jvi.01872-25)
Supplement: Supplemental material — Figure S1; Tables S1 to S3. [file jvi.01872-25-s0001.pdf]

Figure S1

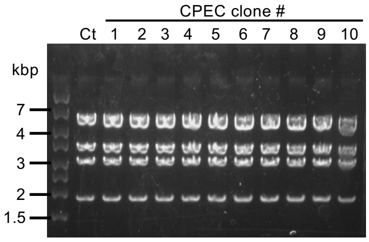

Figure S1. Evaluation of CPEC-derived plasmids coding a full-genome cDNA of RABV.

Gel electrophoresis of CPEC-derived plasmids digested by a restriction enzyme *EcoRI*. Ct: control parental plasmid pCVS.

Table S1

Table S1. Primers used for the preparation of CPER DNA fragments in this study

Target virus	Fragment	Primer name	Type[a]	Sequence (5'-3')
CVS	CVS-N	CVS-CP-N-F	F	TAATACGACTCACTATAGGGACGCTTAACAACAAAACCAGAGAAGAAAAAGACAG
		CVS-CP-N-R	R	CATATTTGGGATGGTTTGAAAGGAGGAGTGTTAGTTTTTTTCATGATGGATATATAC
	CVS-P	CVS-CP-P-F	F	GTATATATCCATCATGAAAAAAACTAACACTCCTCCTTTCAAACCATCCCAAATATG
		CVS-CP-P-R	R	AACGTTCATTTTATCAGTGGTGTTGCCTGTTTTTTTCATGTCTACTCCATAAC
	CVS-M	CVS-CP-M-F	F	GTTATGGAGTAGACATGAAAAAAACAGGCAACACCACTGATAAAATGAACGTT
		CVS-CP-M-R	R	CCTTAAGTCTTTTGAGGGATGTTAATAGTTTTTTTCACATCCAAGAGGCTCAA
	CVS-G	CVS-CP-G-F	F	TTGAGCCTCTTGGATGTGAAAAAAACTATTAACATCCCTCAAAAGACTTAAGG
		CVS-CP-G-R	R	GTTCTTTTGGTCTACAGTTTTTTTCTCGACTGAAATGCTTAG
	CVS-L	CVS-CP-L-F	F	CTAAGCATTTCAGTCGAGAAAAAAACTGTAGACCAAAAGAAC
		CVS-CP-L-R	R	GTCCGGATTCAAGATCTTGTTTTTTTCAAGATGCATCATACAAGA
	CVS-Linker	CVS-CP_Linker-F	F	TCTTGTATGATGCATCTTGAAAAAAACAAGATCTTGAATCCGGAC
		CVS-CP_Linker-R	R	CTGTCTTTTTCTTCTCTGGTTTTGTTGTTAAGCGTCCCTATAGTGAGTCGTATTA
CVS-mCherry (N-P)	CVS-N-mCherry	CVS-CP-N-F	F	TAATACGACTCACTATAGGGACGCTTAACAACAAAACCAGAGAAGAAAAAGACAG
		CVS_CPER1-R	R	GTTTGAAAGGAGGAGTGTTAGTTTTTTTCATGATGGATATATACAATCT
	CVS-mCherry	CVS_NP_mCherry-F	F	TGTATATATCCATCATGAAAAAAACTAACACTCCTCCTTTCAAACCATCCCAAATCGCCACCATGGTGAGCAAGGGCGAGGAG
		CVS_NP_mCherry-R	R	ATTTGGGATGGTTTGAAAGGAGGAGTGTTAGTTTTTTTCACTTGTACAGCTCGTCCATGCCGCCGGTG
	mCherry-CVS-P/M/G	CVS_CPER2-F	F	CAAACCATCCCAAATATGAGCAAGATCTTTGTTA
		CVS-CP-G-R	R	GTTCTTTTGGTCTACAGTTTTTTTCTCGACTGAAATGCTTAG
CVS-mCherry (P-fused)	CVS-P-mCherry	CVS-CP-N-F	F	TAATACGACTCACTATAGGGACGCTTAACAACAAAACCAGAGAAGAAAAAGACAG
		CVS_CPER_m1-R	R	TTATCCTCCTCGCCCTTGCTCACCATGCAGGATGTATAGCGATTCAAATCATCTTG
	CVS-Pfusion-mCherry	CVS_P_mCherry-F	F	TGAATCGCTATACATCCTGCATGGTGAGCAAGGGCGAGGAGGATAACATGG
		CVS_P_mCherry-R	R	GGACTGAGTTCGAAAACTCGGTTACTTGTACAGCTCGTCCATGCCGCCGGTGGAGTG
	CVS-mCherry-M/G	CVS_CPER_m2-F	F	GGCATGGACGAGCTGTACAAGTAACCGAGTTTTCGAACTCAGTCCCTCCAGATAATGA
		CVS-CP-G-R	R	GTTCTTTTGGTCTACAGTTTTTTTCTCGACTGAAATGCTTAG
CVS-GFP (N-P)	CVS-N-GFP	CVS-CP-N-F	F	TAATACGACTCACTATAGGGACGCTTAACAACAAAACCAGAGAAGAAAAAGACAG
		CVS_CPER1-R	R	GTTTGAAAGGAGGAGTGTTAGTTTTTTTCATGATGGATATATACAATCT
	CVS-GFP	CVS_NP_AcGFP-F	F	TGTATATATCCATCATGAAAAAAACTAACACTCCTCCTTTCAAACCATCCCAAATCGCCACCATGGTGAGCAAGGGCGCCGAGCTGT
		CVS_NP_AcGFP-R	R	TAACAAAGATCTTGCTCATATTTGGGATGGTTTGAAAGGAGGAGTGTTAGTTTTTTTCATTACTTGTACAGCTCATCCATGCCGTG
	GFP-CVS-P	CVS_CPER2-F	F	CAAACCATCCCAAATATGAGCAAGATCTTTGTTA
		CVS-CP-P-R	R	AACGTTCATTTTATCAGTGGTGTTGCCTGTTTTTTTCATGTCTACTCCATAAC
HEP	HEP-N	HEP-CP-N-F	F	ACGCTTAACAACAAAACCAAAGAAGAAGCAGACATCGTCAGTTGCAAAGCAAAAA
		HEP-CP-N-R	R	ACTTGGGATGGTTCGAAAGGAGGAGTGTTAGTTTTTTTCA
	HEP-P	HEP-CP-P-F	F	TGAAAAAAACTAACACTCCTCCTTTCGAACCATCCCAAGT
		HEP-CP-P-R	R	GTTCATTTTATTAGTGGTGTTGCCTGTTTTTTTTCATATCGACTCCAT
	HEP-M	HEP-CP-M-F	F	ATGGAGTCGATATGAAAAAAAACAGGCAACACCACTAATAAAATGAAC
		HEP-CP-M-R	R	AGGAACCATCTTTCCTTAAGTCTTTTGAGGGATGTTAATAGTTTTTTTCACAT
	HEP-G	HEP-CP-G-F	F	ATGTGAAAAAAACTATTAACATCCCTCAAAAGACTTAAGGAAAGATGGTTCCT
		HEP-CP-G-R	R	TCCCGGATCCAGCATCTTGATATGGGTCTCGAGATGAGAAGT
	HEP-L	HEP-CP-L-F	F	ACTTCTCATCTCGAGACCCATATCAAGATGCTGGATCCGGGA
		HEP-CP-L-R	R	GATTCAAGATCTTGTTTTTTTCAAGATGCATCATACAAGA
	HEP-Linker	HEP-CP_Linker-F	F	TCTTGTATGATGCATCTTGAAAAAAACAAGATCTTGAATC
		HEP-CP_Linker-R	R	TTTTTGCTTTGCAACTGACGATGTCTGCTTCTTCTTTGGTTTTGTTGTTAAGCGT
CVS-hepG	CVS-hepG-M	CVS-CP-M-F	F	GTTATGGAGTAGACATGAAAAAAACAGGCAACACCACTGATAAAATGAACGTT
		cvsM-R-hepG	R	CCTTAAGTCTTTTGAGGGATGTTAATAGTTTTTTTCACATCCAAGAGGCTCAA
	CVS-hepG-G	cvsM-F-hepG	F	TTGAGCCTCTTGGATGTGAAAAAAACTATTAACATCCCTCAAAAGACTTAAGG
		hepG-R-cvsL	R	GTTCTTTTGGTCTACAGTTTTTTTCTCGACTGAAATGCTTAG
	CVS-hepG-L	hepG-F-cvsL	F	CTAAGCATTTCAGTCGAGAAAAAAACTGTAGACCAAAAGAAC
		CVS-CP-L-R	R	GTCCGGATTCAAGATCTTGTTTTTTTCAAGATGCATCATACAAGA
HEP-cvsG	HEP-cvsG-M	HEP-CP-M-F	F	ATGGAGTCGATATGAAAAAAAACAGGCAACACCACTAATAAAATGAAC
		hepM-R-cvsG	R	CCTTAAGTCTTTTGAGGGATGTTAATAGTTTTTTTCACATCCAAGAGGCTCAA
	HEP-cvsG-G	hepM-F-cvsG	F	TTGAGCCTCTTGGATGTGAAAAAAACTATTAACATCCCTCAAAAGACTTAAGG
		cvsG-R-hepL	R	GTTCTTTTGGTATACAGTTTTTTTCTCGACTGAAATGCTTAG
	HEP-cvsG-L	cvsG-F-hepL	F	CTAAGCATTTCAGTCGAGAAAAAAACTGTATACCAAAAGAAC
		HEP-CP-L-R	R	GATTCAAGATCTTGTTTTTTTCAAGATGCATCATACAAGA

[a]F; forward primer, R; reverse primer

Table S2

Table S2. Mutations and frequency (%) in CPER-derived virus determined by NGS analysis

Genome region		N			IGR	P	IGR	G		L				
Nucleotide position		139	1182	1247	1484	1919	2410	3824	4750	6020	6333	8995	9616	10293
Reference nucleotide		G	C	G	T	C	G	C	G	C	C	T	G	C
Nucleotide change		A	T	T	C	A	T	T	T	T	A	C	T	T
Amino acid change		=	A>V	D>Y	N/A	P>T	N/A	H>Y	M>I	T>I	N>K	=	G>W	=
CPER1	5 dpt							47.1					17.4	
	7 dpt			29.3	10.4			14.4	13.5	10.5			31.2	11.3
	9 dpt			39.4					12.8	11.3			36.7	
	11 dpt			47.2				10.2	11.2				45.4	
CPER2	5 dpt													
	7 dpt		11.4											
	9 dpt													
	11 dpt													
CPER3	5 dpt	40.6				46.8								
	7 dpt	26.7				47.3								
	9 dpt	16.9				55.5								
	11 dpt	13.6				57.1								
CPER4	5 dpt													
	7 dpt													
	9 dpt													
	11 dpt													
CPER5	5 dpt													
	7 dpt													
	9 dpt						11.4							
	11 dpt						10.4				12.8	10.8		

Table S3

Table S3. Primers used for library preparation for the NGS analysis

Primers	Sense	Sequence (5' - 3')	Anealing site positions [a]
CVS-frag1(+)	+	ATACGACTCACTATAGGGACGCTTAACAACAAAACCAGAG	1-22
CVS-frag1(-)	-	GTCATATGGGTCCAAATCTGTCACACTTGGGGATATGATA	3814-3775
CVS-frag2(+)	+	GGTCTGAAGAGGACAAAGACTCTTCTCTGCTTCTAGAATA	3064-3103
CVS-frag2(-)	-	CATTATATTGGCGAGGTTGACTATTTGGTCGTTAGAGATG	7893-7854
CVS-frag3(+)	+	AGAGTTTTTGAAATCTATAGACCTCGGAGGATTGCCAGAT	6972-7011
CVS-frag3(-)	-	CAAGATGCATCATACAAGAATTTTAGCATGCACAGGCTTT	11850-11811

a: Based on the genome nucleotide number of rabies virus CVS strain (GenBank accession No.LC325820.1).
